# Xuebijing injection reduces 28-day mortality in patients with septic shock

**DOI:** 10.1186/s13054-025-05666-3

**Published:** 2025-10-06

**Authors:** Jing Kang, Dingji Hu, Yixuan Li, Lufeng Ma, Hui Chen, Jianfeng Xie, Yi Yang, Tianyi Yang, Zhiqiao Feng, Yan Liu, Haibo Qiu, Songqiao Liu, Chi Zhang

**Affiliations:** 1 Dongzhimen Hospital, Beijing University of Chinese Medicine, Dongcheng District, 100010 Beijing, China; 2 Jiangsu Provincial Key Laboratory of Critical Care Medicine, Department of Critical Care Medicine, Zhongda Hospital, School of Medicine, Southeast University, Jiangsu 210009 Nanjing, China; 3 School of Chinese Medicine, Hong Kong Baptist University, Kowloon Tong, Hong Kong, China; 4 Tianjin Chasesun Pharmaceutical Co., Ltd., Wuqing Development Zone, 301700 Tianjin, China; 5 Institute for Health, Health Care Policy and Aging Research, Rutgers, The State University of New Jersey, New Jersey NJ 08901 New Brunswick, United States; 6 The First People’s Hospital of Lianyungang, The Affiliated Lianyungang Hospital of Xuzhou Medical University, Jiangsu 222000 Lianyungang, China; 7 Institute for Brain Disorders, Beijing University of Chinese Medicine, Dongcheng District, 100010 Beijing, China

Over the past decade, more than 80% of randomized controlled trials (RCTs) in septic shock have failed to identify effective pharmacologic therapies (Supplemental Content 1) [1], underscoring the urgent need for effective treatments. Xuebijing (XBJ) has demonstrated promising efficacy in two recent large-scale RCTs, the EXIT-SEP (*n* = 1817) [2] and XBJ-SCAP (*n* = 710) [3] trials, by significantly reducing 28-day mortality among patients with sepsis or severe community-acquired pneumonia. However, neither trial specifically targeted patients with septic shock, leaving it uncertain whether XBJ provides a survival benefit in this high-risk subgroup. To address this gap, we conducted a harmonized individual patient-level analysis of both trials (Table S1) to evaluate the association between XBJ treatment and mortality in septic shock.

We reanalyzed patient-level data from EXIT-SEP (NCT03238742) and XBJ-SCAP (ChiCTR-TRC-13003534) to construct a harmonized cohort of patients meeting Sepsis-3 criteria for septic shock. Trial elements, including eligibility criteria, treatment strategies, and follow-up periods, were aligned to ensure consistency across studies (Table S1). Septic shock was defined as infection with a vasopressor requirement to maintain mean arterial pressure ≥ 65 mmHg and a lactate concentration >2 mmol/L at baseline [4]. Patients were randomized to receive either intravenous XBJ (100 mL with saline every 12 h for 5 days) or matching placebo, in addition to standard care. The primary outcome was 28-day all-cause mortality; secondary outcomes included ventilator-free and ICU-free days.

A total of 869 patients fulfilled eligibility criteria (432 randomized to XBJ and 437 to placebo). Eight patients (5 XBJ, 3 placebo) were lost to follow-up at day 28, leaving 861 for complete outcome analysis (Figure S1). Baseline characteristics showed no significant imbalances between groups. Selected variables are shown in Fig. 1A, with the full set of baseline data available in the Supplementary Material (Table S2, S3).

At 28 days, 99 of 427 patients in the XBJ group (23.2%) and 132 of 434 in the placebo group (30.4%) had died, corresponding to an absolute risk reduction of 7.2% (95% CI, 1.3–13.1; *p* = 0.02) (Fig. 1B, Table S4). Patients treated with XBJ had more ventilator-free days (mean difference, 2.0; 95% CI, 0.5–3.5; *p* = 0.01) and ICU-free days (mean difference, 1.7; 95% CI, 0.4–3.0; *p* = 0.02). Additionally, improvement in organ dysfunction, as measured by the SOFA score, was more pronounced by day 6 in the XBJ group (mean difference, − 1.0; 95% CI, − 1.5 to − 0.5; *p* < 0.001), indicating enhanced recovery from critical illness (Table S4). Other secondary outcomes, including ICU mortality, hospital length of stay, and APACHE II score changes, did not differ significantly between groups (Table S4). Serious adverse event rates were similar between groups, suggesting no excess safety risk associated with XBJ administration (Table S5). A sensitivity analysis using multiple imputation confirmed the robustness of the result (XBJ 23.2% vs. placebo 30.3%; risk difference, 7.2%; 95% CI, 1.3–13.1; *p* = 0.02, Table S6).

This pooled analysis provides the first RCT-based evidence suggesting that XBJ may offer a clinically meaningful survival benefit in patients with septic shock, a condition with limited therapeutic advances to date. In our harmonized cohort, XBJ was associated with lower 28-day mortality, together with improvements in key secondary outcomes such as ventilator-free and ICU-free days. The potential mechanisms underlying these benefits are supported by experimental and translational data. XBJ has been shown to attenuate inflammatory cytokine release, modulate coagulation pathways, and improve microcirculatory function. Active constituents such as paeoniflorin and hydroxysafflor yellow A may reduce neutrophil recruitment, inhibit gasdermin D–mediated pyroptosis, and limit neutrophil extracellular trap formation, thereby mitigating sepsis-induced organ injury [5]. Together, these mechanisms provide a rational basis for the reduced mortality and organ dysfunction observed in our trial cohort.

Although originally designed for broader sepsis populations, the two trials analyzed in this study allowed us to reconstruct a focused evaluation of XBJ in patients with septic shock by harmonizing definitions and aligning treatment protocols. The preserved randomization and consistent treatment frameworks across both trials minimized confounding and strengthened the validity of the observed treatment effects in this high-risk subgroup. Our pooled cohort of 861 patients with septic shock constitutes one of the largest trial-based datasets for this condition, allowing for well-powered comparisons. Several limitations should be considered. First, the baseline disease severity in the included trials was relatively lower than that typically encountered in unselected ICU populations with septic shock, which may affect the generalizability of our findings. Second, both trials were conducted exclusively in China, underscoring the need for external validation in more diverse international cohorts. An ongoing large real-world registry study (NCT07069894) will be essential for evaluating the effectiveness of XBJ across varied patient populations and clinical settings. Third, this was a post hoc pooled analysis; although randomization was preserved within each trial, the harmonized analysis itself was not prespecified.

These findings indicate that XBJ could be a potential therapeutic option for patients with septic shock. Rigorous confirmation in international and prospective trials is warranted to validate these results.

**Fig. 1:**
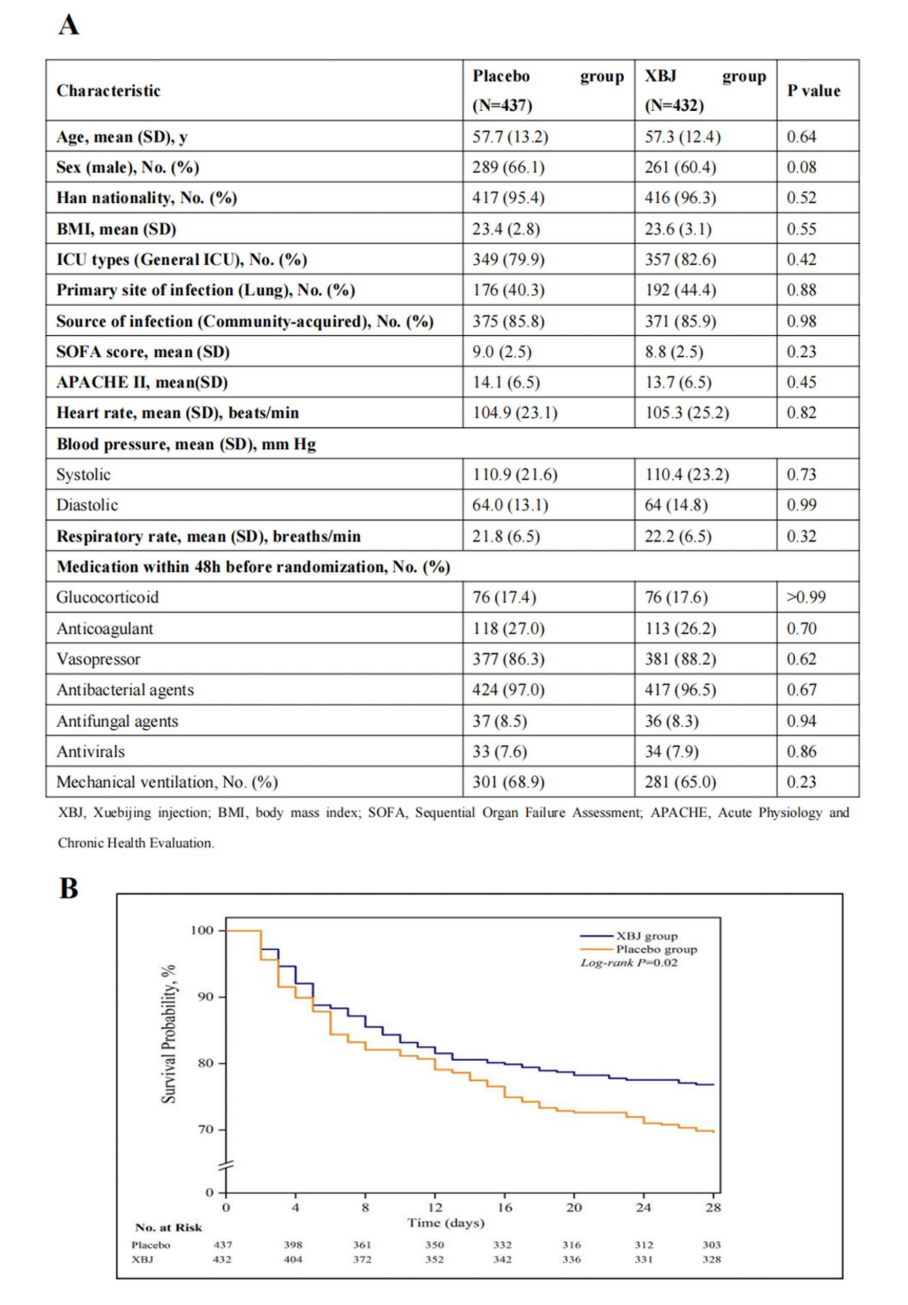
**A** Selected baseline characteristics of patients randomized to XBJ or placebo (full details in Table S2). **B** Kaplan–Meier estimates of survival through day 28

## Supplementary Information

Supplementary Material 1.

## Data Availability

No individual, de-identified participant data (including data dictionaries) from this study will be shared. Researchers may request access to this data for non-commercial, scientific purposes by contacting the corresponding author. Approval for data access will be granted based on the relevance of the proposed analysis to the study objectives, and no specific data repository is used.

## Abbreviations

CI: Confidence interval
ICU: Intensive care unit
SOFA: Sequential organ failure assessment
XBJ: Xuebijing injection
