# Supplementary material for: Xuebijing injection reduces 28-day mortality in patients with septic shock
Source: Crit Care. 2025 Oct 6;29:419. doi: 10.1186/s13054-025-05666-3 (PMC12502413; doi:10.1186/s13054-025-05666-3)

**Supplementary material**

[Supplemental content 1 1](#_Toc1323881487)

[Table. Summary of Clinical Trials on Septic Shock 1](#_Toc398085442)

[Supplemental content 2 4](#_Toc1210463289)

[Table S1. Assessment of comparability across the EXIT-SEP and XBJ-SCAP trials for trial emulation analysis 4](#_Toc1143910192)

[Table S2. Baseline characteristics of the study population 5](#_Toc1424989000)

[Table S3. Use of Other Medications for Sepsis During Study, N(%)^a^ 9](#_Toc1052491656)

[Table S4. Primary and Secondary Outcomes^a^ 10](#_Toc404462053)

[Table S5. Overall Summary of Adverse Events in Safety Population 12](#_Toc1007982016)

[Table S6. Sensitivity Analyses for the Primary Outcome 14](#_Toc1802735376)

[Figure S1. Flow of Participants. 15](#_Toc1874172556)

**Supplemental content 1**

Table. Summary of Clinical Trials on Septic Shock

|  | **Trials** | **Intervention** | **Diagnostic criteria** | **Sample size** | **Primary outcome** | **Result** |
| --- | --- | --- | --- | --- | --- | --- |
| 1 | Effects of adjunctive milrinone versus placebo on hemodynamics in patients with septic shock: a randomized controlled trial  (2025) | Milrinone VS placebo | Septic shock as defined by SEPSIS III | 32:32 | Change in cardiac output from baseline to 6 h | 0.62 L/min, IQR −0.51 to 1.47 VS 0.13 L/min, IQR −0.59 to 0.46, p = 0.043 |
| 2 | Dexmedetomidine to Reduce Vasopressor Resistance in Refractory Septic Shock: α2 Agonist Dexmedetomidine for REfractory Septic Shock (ADRESS): A Double-Blind Randomized Controlled Pilot Trial  (2025) | Dexmedetomidine VS placebo | Septic shock was defined according to the 2016 revised consensus criteria (Sepsis-3) | 16:16 | MAP response to phenylephrine at 6 hours | 1.26 ± 0.23 vs. 1.45 ± 0.26, p = 0.048 |
| 3 | Efficacy of targeting high mean arterial pressure for older patients with septic shock (OPTPRESS): a multicentre, pragmatic, open-label, randomised controlled trial  (2025) | High-target group (target MAP = 80–85 mmHg) VScontrol group (target MAP = 65–70 mmHg) | aged ≥ 65; Septic shock was defined according to the 2016 revised consensus criteria (Sepsis-3) | 258:260 | 90-day all-cause mortality | 39.3% VS 28.6%, P = 0.012 |
| 4 | Early administration of vitamin C in patients with sepsis or septic shock in emergency departments: a multicenter, double-blind, randomized controlled trial: the C-EASIE trial  (2025) | Vitamin C VS placebo | Suspected infection and a National Early Warning Score (NEWS) ≥ 5 | 151:149 | Average post-baseline patient SOFA score on day 2 to 5 | 1.98 (95% CI 1.69 to 2.32) vs 2.19 (95% CI 1.87 to 2.56),  P = 0.30 |
| 5 | Iloprost for the Treatment of Severe Septic Shock with Persistent Hypoperfusion: A Double-Blind, Randomized Controlled Trial  (2025) | 48-hour intravenous infusion of iloprost VS placebo | Septic shock was defined according to the 2016 revised consensus criteria (Sepsis-3) | 116:122 | Change in SOFA score from baseline to day 7 | -4 [-7 - 7] VS -5 [-8 - 5], p = 0.12 |
| 6 | Dexmedetomidine for Reducing Mortality in Patients With Septic Shock: A Randomized Controlled Trial (DecatSepsis)  (2024) | Dexmedetomidine VS standard care | The definition of septic shock was the start of norepinephrine infusion to maintain mean arterial pressure (MAP) of ≥65 mmHg in the presence of sepsis (≥2 systemic inflammatory response syndrome criteria plus suspicion or confirmation of infection) | 45:45 | All-cause in-hospital  mortality | 17(37.8%) VS 25(55.6%) RR, 0.68; 95% CI, 0.43-1.07, P=0.091 |
| 7 | EVALUATION OF HYDROCORTISONE, VITAMIN C, AND THIAMINE FOR THE TREATMENT OF SEPTIC SHOCK: A RANDOMIZED CONTROLLED TRIAL (THE HYVITS TRIAL)  (2023) | Combined hydrocortisone, vitamin C, and thiamine VS standard care | Septic shock was defined according to the 2016 revised consensus criteria (Sepsis-3) | 53:53 | Hospital mortality evaluated at hospital discharge or at 60 days | 28.3% VS 35.8%, P = 0.41 |
| 8 | Prospective evaluation of the efficacy, safety, and optimal biomarker enrichment strategy for nangibotide, a TREM-1 inhibitor, in patients with septic shock (ASTONISH): a double-blind, randomised, controlled, phase 2b trial  （2023） | Nangibotide low-dose VS high-dose VS placebo | Septic shock was defined according to the 2016 revised consensus criteria (Sepsis-3) | 118:121:116 | Change in SOFA score from baseline to day 5 | -1·45 to 1·87, p=0·80  -0·28 to 3·06, p=0·104 |
| 9 | Early adjunctive methylene blue in patients with septic shock: a randomized controlled trial  (2023) | methylene blue VS 0.9% sodium chloride | Septic shock was defined according to the 2016 revised consensus criteria (Sepsis-3) | 45:46 | Time to vasopressor discontinuation at 28 days. | 69 h [IQR 59-83] vs 94 h [IQR 74-141]; p < 0.001 |
| 10 | Remote ischemic conditioning in septic shock: the RECO-Sepsis randomized clinical trial  （2022） | Remote ischemic conditioning VS standard care | Septic shock was defined according to the 2016 revised consensus criteria (Sepsis-3) | 87:91 | Change in SOFA score from baseline to day 4 | 7.2 [5.2-10.7] VS 7.6 [4.9-10.7]; p = 0.919 |
| 11 | Early administration of hydrocortisone, vitamin C, and thiamine in adult patients with septic shock: a randomized controlled clinical trial  （2022） | Hydrocortisone, vitamin C, and thiamine VS placebo | Septic shock was defined according to the 2016 revised consensus criteria (Sepsis-3) | 203:205 | 90-day mortality. | 39.9% VS 39.0%; p = 0.86 |
| 12 | Restriction of Intravenous Fluid in ICU Patients with Septic Shock  (2022) | Restricted intravenous fluid VS standard intravenous fluid | Septic shock was defined according to the 2016 revised consensus criteria (Sepsis-3) | 770:784 | 90-day mortality. | 42.3% VS 42,1%; p=0.96 |
| 13 | Evaluating Vitamin C in Septic Shock: A Randomized Controlled Trial of Vitamin C Monotherapy  （2022） | Vitamin C VS placebo | Sepsis 3 is combined with the traditional SIRS standard | 60:64 | 28-day mortality. | 26.7% VS 40.6%; p = 0.10 |
| 14 | Effect of Ascorbic Acid, Corticosteroids, and Thiamine on Organ Injury in Septic Shock  America  (2020) | Parenteral ascorbic acid, hydrocortisone, and thiamine VS placebo | Based on partial Sepsis-3 criteria | 101:99 | Change in SOFA score from baseline to day 3 | 9.1 to 4.4 [-4.7] points VS 9.2 to 5.1 [-4.1] points; p = 0.12 |
| 15 | Effect of Vitamin C, Hydrocortisone, and Thiamine vs Hydrocortisone Alone on Time Alive and Free of Vasopressor Support Among Patients With Septic Shock  (2020) | Vitamin C, hydrocortisone, and thiamine VS hydrocortisone | Septic shock was defined according to the 2016 revised consensus criteria (Sepsis-3) | 109:107 | Time alive and vasopressor-free up to day 7 | 122.1 hours (QR, 76.3-145.4 hours) VS 124.6 hours (IQR, 82.1-147.0 hours); p=0.83 |
| 16 | Early Use of Norepinephrine in Septic Shock Resuscitation (CENSER). A Randomized Trial  (2019) | Early norepinephrine administration VS placebo | Surviving Sepsis Campaign: International Guidelines for Management of Severe Sepsis and Septic Shock: 2012 | 155:155 | Shock control rate within 6 hours of diagnosis | 76.1% VS 48.4%; p < 0.001 |
| 17 | Effect of a Resuscitation Strategy Targeting Peripheral Perfusion Status vs Serum Lactate Levels on 28-Day Mortality Among Patients With Septic Shock: The ANDROMEDA-SHOCK Randomized Clinical Trial  (2019) | Peripheral perfusion VS lactate | Septic shock was defined according to the 2016 revised consensus criteria (Sepsis-3) | 212:212 | 28-day mortality. | 34.9% VS 43.4%; p = 0.06 |
| 18 | Hydrocortisone plus Fludrocortisone for Adults with Septic Shock  (2018) | Hydrocortisone fludrocortisone, drotrecogin alfa VS placebo | Based on “Septic shock. Lancet 2005; 365:63-78” | 614:627 | 90-day mortality. | 43.0% VS 49.1%; p = 0.03 |
| 19 | Adjunctive Glucocorticoid Therapy in Patients with Septic Shock  (2018) | Hydrocortisone VS placebo | Based on “Definitions for sepsis and organ failure and guidelines for the use of innovative therapies in sepsis. Chest 1992; 101:1644-1655” | 1832:1826 | 90-day mortality. | 27.9% VS 28.8%; p = 0.05 |
| 20 | Effect of Targeted Polymyxin B Hemoperfusion on 28-Day Mortality in Patients With Septic Shock and Elevated Endotoxin Level  (2018) | Two polymyxin B VS standard therapy | Based on the Sepsis-1 and Sepsis-2 definitions of septic shock and the clinical practice guidelines of that time. | 224:226 | 28-day mortality. | 37.7% VS 34.5%; p = 0.49 |
| **Published in 2016. The Third International Consensus Definitions for Sepsis and Septic Shock (Sepsis-3)** | | | | | | |
| 21 | Effect of Hydrocortisone on Development of Shock Among Patients With Severe Sepsis: The HYPRESS Randomized Clinical Trial  （2016） | Hydrocortisone VS placebo | Based on Clinical evidence of infection and SIRS and organ dysfunction | 190:190 | Septic shock development within 14 days | 21.2% VS 22.9%; p = 0.70 |
| 22 | Trial of early, goal-directed resuscitation for septic shock  (2015) | Early, goal-directed therapy VS usual care | Based on “Definitions for sepsis and organ failure and guidelines for the use of innovative therapies in sepsis. Chest 1992; 101:1644-1655” | 630:630 | 90-day mortality. | 29.5% VS 29.3%; p = 0.90 |
| 23 | Goal-directed resuscitation for patients with early septic shock  (2014) | Early goal-directed therapy VS usual care | Based on “Definitions for sepsis and organ failure and guidelines for the use of innovative therapies in sepsis. Chest 1992; 101:1644-1655” | 796:804 | 90-day mortality. | 18.6% VS 18.8%; p = 0.90 |
| 24 | Albumin replacement in patients with severe sepsis or septic shock  (2014) | Albumin and crystalloid solution VS crystalloid solution | Based on “Definitions for sepsis and organ failure and guidelines for the use of innovative therapies in sepsis. Chest 1992; 101:1644-1655” | 903:907 | 28-day mortality. | 31.8% VS 32.0%; p = 0.94 |

Note: In the diagnostic criteria section, red indicates that the Sepsis 3.0 diagnostic criteria were not used to screen patients, while green indicates that the Sepsis 3.0 criteria were applied. In the sample size section, blue represents a sample size larger than 869 (the sample size of this study), while purple indicates a sample size equal to or smaller than 869. For the primary outcome section, green signifies that 28-day mortality was used as the primary outcome, yellow indicates the use of mortality as the outcome measure but not specifically for 28 days, and red denotes other outcome measures. Finally, in the conclusion section, grey represents negative outcomes, and green represents positive outcomes.

**Supplemental content 2**

**Table S1. Assessment of comparability across the EXIT-SEP and XBJ-SCAP trials for trial emulation analysis**

| Pocock Criteria | EXIT-SEP | XBJ-SCAP | Harmonization Notes |
| --- | --- | --- | --- |
| Same eligibility criteria applied | Adults aged 18–75 with Sepsis-3 and SOFA 2–13; excluded if >48h since diagnosis or with severe comorbidities | Patients with severe community-acquired pneumonia at high risk of sepsis; different inclusion focus, though overlapping | Standardized septic shock definition (Sepsis-3 criteria with vasopressor use and lactate >2 mmol/L) applied retrospectively to both trials. |
| Received a precisely defined treatment | XBJ 100 mL + NS 100 mL every 12h for 5 days | XBJ 100 mL + NS 100 mL every 12h for 5–7 days | Treatment protocol (dose, route, frequency) identical across trials, enabling direct comparison. |
| Same outcome definitions and ascertainment methods | Primary: 28-day all-cause mortality; Secondary: SOFA, APACHE II, ICU stay, MV duration | Primary: 28-day mortality; Secondary: SOFA score, ventilator-free days, ICU/hospital stay | Mortality and organ function outcomes defined similarly; harmonized endpoints selected for analysis. |
| Similar distribution of key baseline characteristics | Included patients across 45 centers in China with similar demographics; strict inclusion criteria ensured balance | Patients had slightly different clinical characteristics; distribution comparable but not identical | After applying unified inclusion/exclusion and aligning analysis population, baseline variables were well balanced. |
| Same institution and clinical investigators | All sites and investigators located in China; centralized training and coordination | Multicenter trial within China; partial overlap in institutions/investigators likely but not fully identical | Both trials conducted in Chinese ICUs under GCP standards; institutional overlap not complete but settings comparable. |

**Table S2. Baseline characteristics of the study population**

| **Characteristic** | **Placebo group (N=437)** | **XBJ group (N=432)** | **P value** |
| --- | --- | --- | --- |
| Age, mean (SD), y | 57.7 (13.2) | 57.3 (12.4) | 0.64 |
| Sex, No. (%) |  |  | 0.08 |
| Men | 289 (66.1) | 261 (60.4) |  |
| Women | 148 (33.9) | 171 (39.6) |  |
| Han nationality, No. (%)^a^ | 417 (95.4) | 416 (96.3) | 0.52 |
| BMI, mean (SD)^b^ | 23.4 (2.8) | 23.6 (3.1) | 0.55 |
| ICU types, No. (%) |  |  | 0.42 |
| General ICU | 349 (79.9) | 357 (82.6) |  |
| Emergency ICU | 75 (17.2) | 68 (15.7) |  |
| Surgical ICU | 12 (2.7) | 7 (1.6) |  |
| Respiratory ICU | 1 (0.2) | 0 |  |
| Primary site of infection, No. (%) |  |  | 0.88 |
| Lung | 176 (40.3) | 192 (44.4) |  |
| Intra-abdominal | 152 (34.8) | 144 (33.3) |  |
| Urinary tract | 34 (7.8) | 32 (7.4) |  |
| Skin or soft tissue | 19 (4.3) | 15 (3.5) |  |
| Central nervous system | 6 (1.4) | 7 (1.6) |  |
| Blood | 5 (1.1) | 3 (0.7) |  |
| Other^c^ | 45 (10.3) | 39 (9.0) |  |
| Source of infection, No. (%) |  |  | 0.98 |
| Community-acquired | 375 (85.8) | 371 (85.9) |  |
| Nosocomial | 62 (14.2) | 61 (14.1) |  |
| Preexisting conditions, No. (%) |  | | |
| Hypertension | 115 (26.3) | 99 (22.9) | 0.24 |
| Diabetes Mellitus | 100 (22.9) | 83 (19.2) | 0.18 |
| Coronary Artery Disease | 28 (6.4) | 30 (6.9) | 0.75 |
| Liver | 22 (5.0) | 14 (3.2) | 0.18 |
| Stroke | 16 (3.7) | 19 (4.4) | 0.58 |
| Renal | 9 (2.1) | 11 (2.5) | 0.63 |
| COPD | 5 (1.1) | 7 (1.6) | 0.55 |
| Malignancy | 4 (0.9) | 6 (1.4) | 0.54 |
| Other | 55 (12.6) | 56 (13.0) | 0.86 |
| SOFA score, mean (SD)^d^ | 9.0 (2.5) | 8.8 (2.5) | 0.23 |
| Organ dysfunction, No. (%)^e^ |  | | |
| Cardiovascular | 374 (85.6) | 370 (85.6) | 0.98 |
| Respiratory | 333 (76.2) | 341 (78.9) | 0.33 |
| Coagulation | 156 (35.7) | 129 (29.9) | 0.07 |
| Hepatic | 106 (24.3) | 102 (23.6) | 0.82 |
| Neurologic | 157 (35.9) | 150 (34.7) | 0.71 |
| Renal | 93 (21.3) | 84 (19.4) | 0.50 |
| APACHE II, mean(SD)^f^ | 14.1 (6.5) | 13.7 (6.5) | 0.45 |
| <25 | 411 (94.1) | 407 (94.2) | 0.92 |
| ≥25 | 26 (6.0) | 25 (5.8) |  |
| Heart rate, mean (SD), beats/min | 104.9 (23.1) | 105.3 (25.2) | 0.82 |
| Respiratory rate, mean (SD), breaths/min | 21.8 (6.5) | 22.2 (6.5) | 0.32 |
| Blood pressure, mean (SD), mm Hg |  | | |
| Systolic | 110.9 (21.6) | 110.4 (23.2) | 0.73 |
| Diastolic | 64.0 (13.1) | 64.0 (14.8) | 0.99 |
| Medication within 48h before randomization, No. (%)^g^ |  | | |
| Glucocorticoid | 76 (17.4) | 76 (17.6) | >0.99 |
| Anticoagulant | 118 (27.0) | 113 (26.2) | 0.70 |
| Vasopressor | 377 (86.3) | 381 (88.2) | 0.62 |
| Antimicrobial agents |  | | |
| Antibacterial agents | 424 (97.0) | 417 (96.5) | 0.67 |
| Antifungal agents | 37 (8.5) | 36 (8.3) | 0.94 |
| Antivirals | 33 (7.6) | 34 (7.9) | 0.86 |
| Mechanical ventilation, No. (%) | 301 (68.9) | 281 (65.0) | 0.23 |
| PaO2/FiO_2_ ratio (SD) | 234.9 (118.9) | 222.6 (108.3) | 0.11 |
| Creatinine (µmol/L) | 119.5 (70.4) | 119.2 (86.3) | 0.97 |
| Culture-proven pathogens, No. (%) |  | | |
| Gram negative | 164 (37.5) | 159 (36.8) | 0.83 |
| Gram positive | 70 (16.0) | 65 (15.0) | 0.69 |
| Gram positive and negative | 31 (7.1) | 21 (4.9) | 0.17 |
| Fungal | 40 (9.2) | 36 (8.3) | 0.67 |
| Virials | 7 (1.6) | 5 (1.2) | 0.57 |
| Atypical pathogens | 0 (0.0) | 1 (0.2) | 0.50 |

Abbreviations: XBJ, Xuebijing injection; BMI, body mass index; COPD, Chronic Obstructive Pulmonary Disease; SOFA, Sequential Organ Failure Assessment; APACHE, Acute Physiology and Chronic Health Evaluation.

^a^Race was reported by the patient.

^b^Calculated as weight in kilograms divided by height in meters squared.

^c^ Other site of infection included unknown source.

^d^The SOFA score includes sub-scores ranging from 0 to 4 for each of six components (respiratory, coagulation, liver, cardiovascular, neurologic, and renal components), with higher scores indicating more severe organ dysfunction.

^e^Organ dysfunctions were defined as a SOFA score of 2 or higher for each of six components.

^f^APACHE II scores range from 0 to 71; 0 indicates the lowest prediction of mortality and 71 indicates the highest. Data on APACHE II scores were available for 195 patients in the placebo group and 172 in the XBJ group.

^g^ Data on glucocorticoid or anticoagulant or vasopressor were available for 415 patients in the placebo group and 415 in the XBJ group.

**Table S3. Use of Other Medications for Sepsis During Study, N(%)^a^**

|  | **Placebo group (N=415)** | **XBJ group (N=415)** |
| --- | --- | --- |
| Glucocorticoid | 127 (30.6) | 127 (30.6) |
| Anticoagulant | 184 (44.3) | 211 (50.8) |
| Vasopressors | 397 (95.7) | 398 (95.9) |
| Antimicrobials |  |  |
| Antibacterial agents | 414 (99.8) | 414 (99.8) |
| Carbapenems | 282 (68.0) | 291 (70.1) |
| Other beta-lactams | 281 (67.7) | 258 (62.2) |
| Quinolones | 81 (19.5) | 111 (26.7) |
| Oxazolidinones | 88 (21.2) | 87 (21.0) |
| Glycylcyclones | 83 (20.0) | 81 (19.5) |
| Glycopeptide | 72 (17.3) | 68 (16.4) |
| Aminoglycoside | 31 (7.5) | 22 (5.3) |
| Cyclic lipopeptide | 5 (1.2) | 13 (3.1) |
| Macrolide | 6 (1.4) | 5 (1.2) |
| Other^b^ | 101 (24.3) | 84 (20.2) |
| Antifungal agents | 111 (26.7) | 110 (26.5) |
| Antivirals | 40 (9.6) | 42 (10.1) |

^a^ Medications used during study was only collected for patients receiving the study drug in the placebo group (n=415) and in the XBJ group (n=415) from the EXIT-SEP study.

^b^ Other antibacterial agents included Nitroimidazoles, Sulfanilamide, Polypeptides, Cyclic lipopeptide, Tetracyclines Fosfomycin, Polypeptides, Rifamycin, Anti Mycobacterium drugs, Lincomamides, Chloramphenicols, Furan, Anti tuberculosis drugs, and Rifamycin derivatives.

**Table S4. Primary and Secondary Outcomes^a^**

| **Variable** | **Placebo group (N=437)** | **XBJ group (N=432)** | **Difference (95%CI)** | ***P* Value** |
| --- | --- | --- | --- | --- |
| **Primary outcome**^b^ |  |  |  |  |
| 28-day mortality, No./total No. (%) | 132/434 (30.4) | 99/427 (23.2) | 7.2 (1.3 to 13.1) | 0.02 |
| **Secondary outcomes**^c^ |  |  |  |  |
| Mortality, No./total No. (%) |  |  |  |  |
| ICU | 101/434 (23.3) | 80/427 (18.7) | 4.5 (-0.8 to 10.0) | 0.10 |
| Hospital | 119/434 (27.4) | 91/427 (21.3) | 6.1 (0.004 to 11.8) | 0.04 |
| Length of stay, mean(95%CI), d^d^ |  |  |  |  |
| In ICU | 11.2 (10.5 to 11.9) | 10.6 (9.8 to 11.3) | 0.6 (-0.4 to 1.7) | 0.25 |
| In Hospital ^e^ | 16.2 (15.4 to 17.1) | 15.6 (14.8 to 16.5) | 0.6 (-0.6 to 1.8) | 0.32 |
| 28-day cumulative mechanical ventilation-free days, mean (95%CI), d^f^ | 15.2 (14.1 to 16.2) | 17.1 (16.1 to 18.2) | -2.0 (-3.5 to -0.5) | 0.01 |
| 28-day ICU-free days, mean (95%CI), d^g^ | 11.0 (10.0 to 11.9) | 12.7 (11.7 to 13.6) | -1.7 (-3.0 to -0.4) | 0.02 |
| Change, SOFA score, mean (95%CI)^h^ |  |  |  |  |
| 3-day | -1.2 (-1.5 to -0.9) | -1.3 (-1.6 to -0.9) | 0.1 (-0.4 to 0.5) | 0.76 |
| 6-day | -2.4 (-2.7 to -2.0) | -3.3 (-3.7 to -3.0) | 1.0 (0.5 to 1.5) | <0.001 |
| Change, APACHE II score, mean (95%CI)^h^ |  |  |  |  |
| 3-day | -2.4 (-2.9 to -2.0) | -2.3 (-2.8 to -1.9) | -0.1 (-0.7 to 0.6) | 0.80 |
| 6-day | -2.7 (-3.2 to -2.1) | -3.3 (-3.9 to -2.8) | 0.6 (-0.1 to 1.4) | 0.10 |

^a^For rows including number/total number, the total number refers to the number of patients with valid data.

^b^A total of 8 patients were not collected due to lost follow-up.

^c^Missing data not imputed for secondary outcomes analyses.

^d^Data were calculated using a mixed-effects model.

^e^The length of stay in the hospital included the length of stay in the ICU.

^f^The mechanical ventilation-free days were defined as the total number of days a patient was alive and not on mechanical ventilation from randomization to 28 days. Data were calculated using a generalized linear model.

^g^The ICU-free days were defined as the number of days alive and free of ICU from randomization to 28 days. Data were calculated using a generalized linear model.

^h^Negative changes indicate better outcomes. Data were calculated using a repeated-measures mixed-effects model. Data were only from the EXIT-SEP study.

**Table S5. Overall Summary of Adverse Events in Safety Population**

|  | **Placebo group (N=437)** | **XBJ group (N=432)** |
| --- | --- | --- |
| **Any SAE^a^** | 11 (2.7) | 7 (1.7) |
| **Cardiac disorders** | 9 (2.2) | 7 (1.7) |
| Atrial fibrillation | 5 (1.2) | 4 (1.0) |
| Myocardial Infarction | 2 (0.5) | 1 (0.2) |
| Arrhythmia ventricular | 1 (0.2) | 1 (0.2) |
| Arrhythmic storm | 1 (0.2) | 1 (0.2) |
| Blood and lymphatic system disorders | 1 (0.2) | 0 (0.0) |
| DIC | 1 (0.2) | 0 (0.0) |
| **Gastrointestinal disorders** | 1 (0.2) | 0 (0.0) |
| Upper gastrointestinal hemorrhage | 1 (0.2) | 0 (0.0) |
| **Nervous system disorders** | 1 (0.2) | 0 (0.0) |
| Brain infarction | 1 (0.2) | 0 (0.0) |
| **Any AE** | 125 (28.6) | 112 (25.9) |
| **Investigations** | 125 (28.6) | 110 (25.5) |
| Haemoglobin decreased | 20 (4.6) | 25 (5.8) |
| ALT increased | 22 (5.0) | 22 (5.1) |
| Platelet count decreased | 23 (5.3) | 18 (4.2) |
| White blood cell count increased | 19 (4.3) | 20 (4.6) |
| BUN increased | 19 (4.3) | 15 (3.5) |
| Bilirubins total increased | 17 (3.9) | 12 (2.8) |
| Glutamic-oxaloacetic transaminase increased | 12 (2.7) | 17 (3.9) |
| APTT prolonged | 12 (2.7) | 10 (2.3) |
| Blood creatinine increased | 10 (2.3) | 5 (1.2) |
| Fibrinogen increased | 13 (3.0) | 2 (0.5) |
| Prothrombin time increased | 7 (1.6) | 5 (1.2) |
| Blood creatinine decreased | 4 (0.9) | 6 (1.4) |
| Fibrin D dimer increased | 5 (1.1) | 5 (1.2) |
| Urine white blood cell increased | 2 (0.5) | 8 (1.9) |
| Sugar blood increased | 4 (0.9) | 5 (1.2) |
| Urine red blood cells increased | 6 (1.4) | 3 (0.7) |
| Decreased white cell count | 3 (0.7) | 4 (0.9) |
| Glucose urine elevated | 5 (1.1) | 1 (0.2) |
| Platelet count increased | 2 (0.5) | 4 (0.9) |
| Faecal occult blood positive | 2 (0.5) | 2 (0.5) |
| Fibrinogen decreased | 2 (0.5) | 2 (0.5) |
| Urinary protein increased | 1 (0.2) | 3 (0.7) |
| BUN decreased | 1 (0.2) | 1 (0.2) |
| Blood pressure increased | 0 (0.0) | 2 (0.5) |
| AST decreased | 0 (0.0) | 1 (0.2) |
| Bilirubin total increased | 0 (0.0) | 1 (0.2) |
| Haemoglobin increased | 0 (0.0) | 1 (0.2) |
| **General symptoms or Gastrointestinal disorders** | 1 (0.2) | 1 (0.2) |
| Fever | 1 (0.2) | 1 (0.2) |
| Gastrointestinal disorders | 0 (0.0) | 1 (0.2) |
| Emesis | 0 (0.0) | 1 (0.2) |
| **Eye disorders** | 0 (0.0) | 1 (0.2) |
| Proptosis | 0 (0.0) | 1 (0.2) |
| **Musculoskeletal and connective tissue disorders** | 0 (0.0) | 1 (0.2) |
| Muscle twitching | 0 (0.0) | 1 (0.2) |
| **Psychiatric disorders** | 0 (0.0) | 1 (0.2) |
| Restless | 0 (0.0) | 1 (0.2) |

^a^ Data were only from the EXIT-SEP study.

**Table S6. Sensitivity Analyses for the Primary Outcome**

| **28-day mortality** | **Placebo group (N=201)** | **XBJ group (N=177)** | **Difference (95%CI)** | ***P* Value** |
| --- | --- | --- | --- | --- |
| Model 1 | 30.3 (26.1 to 34.7) | 23.2 (19.2 to 27.2) | 7.2 (1.3 to 13.1) | 0.02 |

We had 8 cases with missing data from the study 1. Multiple imputation was used to impute missing values under the missing-at random assumption (MAR). Specifically, 100 imputed data sets were generated using the fully conditional specification method with the number of iterations set to 10 for the following variables: group (XBJ and Placebo), and response variable (28-day mortality: yes, no). After multiple imputation, each of the hundred multiple imputation datasets was analyzed by generalized linear model. The overall estimates were calculated using Rubins rules. The multiple imputation procedure (PROC MI) in SAS, version 9.4 was used.

**Figure S1. Flow of Participants.**


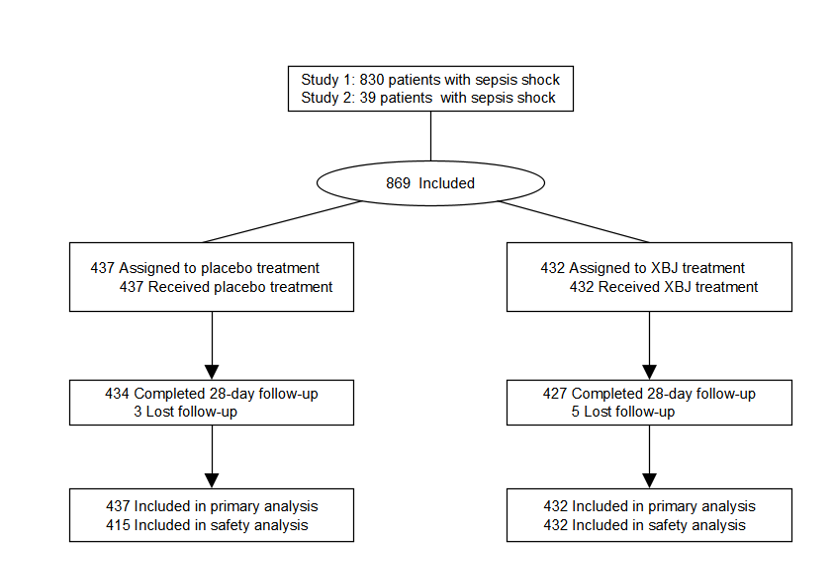

Supplement: Supplementary file 1 — Supplementary Material 1. [file 13054_2025_5666_MOESM1_ESM.docx]
